# Knowledge, Attitudes and Practices Towards COVID-19 Amongst Ethnic Minorities in Hong Kong

**DOI:** 10.3390/ijerph17217878

**Published:** 2020-10-27

**Authors:** Cho Lee Wong, Jieling Chen, Ka Ming Chow, Bernard M.H. Law, Dorothy N.S. Chan, Winnie K.W. So, Alice W.Y. Leung, Carmen W.H. Chan

**Affiliations:** The Nethersole School of Nursing, Faculty of Medicine, The Chinese University of Hong Kong, Hong Kong, China; jojowong@cuhk.edu.hk (C.L.W.); elainechan9@163.com (J.C.); bernardlaw@cuhk.edu.hk (B.M.H.L.); dorothycns@cuhk.edu.hk (D.N.S.C.); winnieso@cuhk.edu.hk (W.K.W.S.); alicewyleung@cuhk.edu.hk (A.W.Y.L.); whchan@cuhk.edu.hk (C.W.H.C.)

**Keywords:** COVID-19, knowledge, attitudes, practices, ethnic minorities, Hong Kong

## Abstract

This study assessed the knowledge, attitudes and practices (KAP) towards coronavirus disease 2019 (COVID-19) among South Asians in Hong Kong and examined the factors that affect KAP towards COVID-19 in this population. This cross-sectional descriptive study recruited participants with assistance from South Asian community centres and organisations. A total of 352 participants completed questionnaires to assess their level of KAP towards COVID-19. The mean knowledge score was 5.38/10, indicating a relatively low knowledge level. The participants expressed certain misconceptions regarding the prevention of COVID-19 infection. They perceived a mild risk related to the disease, had positive attitudes regarding its prevention and often implemented recommended disease-preventive measures, such as maintaining social distance (88.1%) and wearing masks in public (94.3%). Participants who were male, had a secondary school education or lower and who perceived a lower risk of being infected and lower self-efficacy were less likely to implement preventive measures. Culturally and linguistically appropriate health education could be developed to increase the knowledge of South Asians, especially those with lower education levels, about COVID-19 and to encourage them to implement the necessary preventive measures.

## 1. Introduction

Coronavirus disease 2019 (COVID-19), an emerging respiratory disease that was first reported in Wuhan, Hubei Province, China, in December 2019, is caused by the novel severe acute respiratory syndrome coronavirus-2 (SARS-CoV-2). This virus is highly contagious, and symptoms of the related disease include fever, malaise, dry cough and dyspnoea [1]. The mode of transmission of SARS-CoV-2 is primarily through respiratory droplets and direct contact. Its incubation period ranges between 1 and 14 days, with an average of 5 days [1].

The global COVID-19 pandemic is ongoing and has spread quickly, and confirmed cases have been reported in the majority of countries. The World Health Organization (WHO) declared this disease a public health emergency of international concern on 30 January 2020 and encouraged all countries to work together to prevent the worsening of the pandemic [2]. As of 9 September 2020, there have been more than 27 million confirmed cases, resulting in more than 891,000 deaths worldwide [2]. Compared with other countries and regions worldwide, Hong Kong appears to be less severely affected by the COVID-19 pandemic. According to the Center of Health Protection (CHP) [3], almost 5000 cases of COVID-19 have been recorded to date in the region, and 99 patients were reported to have died of the disease.

Currently, no vaccines are available for the effective prevention of SARS-CoV-2 infection. The current COVID-19 treatment regimen is limited to symptom management. Accordingly, the implementation of preventive measures and infection control procedures is of utmost importance to reduce the spread of this disease [1]. Some unprecedented measures have been implemented by the Hong Kong Government to control the spread of infection, including the enactment of regulations to maintain social distancing by prohibiting any group gatherings of more than four people in public places and ordering the closure of public facilities. Other currently enforced measures include the denial of entry of all foreign nationals to Hong Kong, compulsory quarantine for inbound travellers and isolation of infected persons and suspected cases at certain healthcare facilities. In addition, the Government has endeavoured to raise the public’s knowledge and awareness of COVID-19 and its prevention through advertisements and social media [3].

The effectiveness of these implemented COVID-19 preventive measures is largely affected by the knowledge, attitudes and practice (KAP) of the public towards this disease. Knowledge pertains to the community’s comparative level of knowledge about relevant biomedical concepts. Attitude refers to thoughts, feelings and actions about a concept that predispose people to act in a preferential manner. Practice refers to the extent to which preventive measures have been implemented among the public [4]. KAP may vary substantially among population groups according to their cultural and socio-economic characteristics. A study in Egypt demonstrated that individuals who are older, less educated, have a lower income or live in a rural area have a lower level of knowledge about COVID-19 [5]. Another study in China also revealed similar findings [6]. Emerging evidence from Italy, the United Kingdom and the United States indicates that ethnic minorities may experience worse outcomes associated with COVID-19, as evidenced by an overrepresentation of these populations among hospitalised COVID-19 patients [7,8]. Previous research suggested the existence of an interaction between ethnicity and disease spread and progression that is driven by differences in biological, cultural, behavioural and social characteristics, including comorbidities, low socioeconomic status, work conditions, living standards, intergenerational cohabitation and health-seeking behaviours [8]. However, data pertaining to the KAP of ethnic minorities in Hong Kong towards COVID-19 are scarce.

According to the Census and Statistics Department [9], the population of ethnic minority residents in Hong Kong has increased by more than 30% in the past decade. Most ethnic minorities (excluding foreign domestic helpers from Indonesia and the Philippines) originate from South Asian countries, such as India, Pakistan and Nepal. However, only approximately 10% of residents of Pakistani and Nepalese ethnicity have attained post-secondary education, and less than 10% of either population speaks either English or Cantonese in their daily communications [9]. Therefore, South Asian residents of Hong Kong may possess insufficient knowledge about COVID-19 because they are unable to comprehend the primarily Chinese- and English-language information about COVID-19 and its preventive measures as disseminated by the Hong Kong Government. Individuals’ cultural beliefs may also pose remarkable challenges to the effective dissemination of health information [10]. For instance, the fatalistic belief that illness can occur outside of a person’s direct control suggests that some individuals tend to externalise their responsibility to implement preventive measures for the benefit of society [11]. These observations suggest that ethnic minorities generally have a greater need for education on the effective prevention of COVID-19. To effectively control the pandemic in Hong Kong, local ethnic minorities should be provided education with the aim of increasing their awareness about the importance of implementing the Government-recommended infection prevention measures.

A better understanding of the KAP towards COVID-19 held by individuals in South Asian minority populations at the height of the pandemic is needed to ensure the delivery of more effective education on this issue. Moreover, identifying the KAP held by South Asians in Hong Kong towards COVID-19 would help to reveal any misconceptions possessed by these individuals that may reduce their intentions to exhibit health behavioural changes. This study assessed the KAP of South Asians towards COVID-19 and examined the factors that affect their KAP towards the disease.

## 2. Materials and Methods

### 2.1. Study Design and Study Settings

This cross-sectional descriptive study was conducted in a community-based setting in Hong Kong. Participants were recruited via South Asian associations and community centres.

### 2.2. Participants

Web-based and community-based convenience and snowball sampling approaches were applied for subject recruitment. In this regard, we sought support through our close connections with South Asian community centres and organisations, which were developed during collaborations for the implementation of our previous health promotion projects [12]. These organisations were asked to circulate an online questionnaire to be completed by their members. The eligibility criteria for participation in this online survey were (1) South Asian minority status, (2) an age of 18 years or older and (3) an ability to comprehend and communicate in Urdu, Nepali or English.

A sample size of 118 participants was determined to be adequate to detect a medium effect size in a multiple regression model with 10 predictors at a power of 80% and a significance level of 5%.

### 2.3. Measures

The questionnaire was developed by the research team with reference to previous research on KAP toward SARS [13] and in accordance with the current recommendations and guidelines from the United States Centre for Disease Control and Prevention (CDC), WHO and CHP. The primary version was prepared in English and translated into Urdu and Nepali using standard translating procedures. The translated versions were then reviewed by expert panels to ensure semantic and content equivalence. A convenience sample of nine South Asian residents of different ages was recruited to ensure that the contents of the questionnaire would be comprehensible to South Asians.

Knowledge about COVID-19 was assessed using 10 true-or-false question items that aimed to determine the participants’ knowledge about the signs and symptoms, transmission route and prevention measures of COVID-19. One point was awarded for each correct answer, while zero points were given for each item that was answered incorrectly or left unanswered by selecting the response ‘do not know’. The possible knowledge score ranged from 0 to 10, with a higher score indicating a better level of knowledge possessed by the participant.

Attitudes towards COVID-19 were assessed using 10 items that covered two aspects: the participants’ perceived risk of disease (7 items) and their perceived self-efficacy in controlling the disease (3 items). Each item was rated on a 5-point Likert scale ranging from 1 (strongly disagree) to 5 (strongly agree). The mean score of each subscale was calculated to indicate the degrees of participants’ attitudes in the respective domains. In this study, the Cronbach’s alpha for the entire scale was 0.80; the perceived risk of disease subscale was 0.76, and the perceived self-efficacy subscale was 0.76.

Practices of preventive measures against COVID-19 were assessed using 10 items that covered the aspects of personal hygiene practices and maintaining social distance. Each item was rated on a 4-point Likert scale ranging from 1 (never) to 4 (always). A higher score indicated a higher level of implementation of the preventive measures. This portion of the questionnaire received a Cronbach’s alpha of 0.85 in our study.

The author-developed questionnaire was also designed to collect socio-demographic information from the participants during the online survey. This information included age, gender, birthplace, marital status, work status, education level, monthly household income and religion.

### 2.4. Data Collection

The data were collected via self-administered online surveys or face-to-face interviews. An online survey portal was created using SurveyMonkey, a secure and mobile device-based data collection tool. The portal included a brief description of the study, a consent form and the questionnaire. Organisations that consented to collaborate with our team were provided a link to the online questionnaire and were asked to promote our study by circulating the link among their members. Potential participants who were interested in the study were invited to access the link and the online questionnaire via their personal electronic devices, and were asked via the portal to provide informed consent to participate in the study and to complete the online questionnaire. Our research assistants also invited the members of South Asian community centres and organisations to complete the questionnaire via face-to-face interviews. The questionnaire took about 10 min to complete.

### 2.5. Statistical Analysis

IBM SPSS Statistics for Windows, version 25.0 (IBM Corp., Armonk, NY, USA) was used for the statistical analysis. Descriptive statistics, such as means and standard deviations for continuous variables and proportions for categorical variables, were used to summarise the participants’ socio-demographic characteristics. The frequencies of correct knowledge answers and various attitudes and practices were reported. The associations of demographic factors with KAP were examined using the F test for categorical variables and Pearson correlation for continuous variables. A Pearson correlation was used to examine relationships among KAP. Additionally, a multivariable linear regression analysis was conducted to identify the factors that affected the participants’ practices of preventive measures, using demographic factors, knowledge and attitudes as the independent variables and practices as the outcome variable. All statistical tests were two-tailed, and the statistical significance level was set at *p* < 0.05.

### 2.6. Ethical Considerations

Ethical approval was obtained from the Survey and Behavioural Research Committee of the Chinese University of Hong Kong. The survey did not collect identifiable data. A consent form was prepared in three languages (English, Urdu and Nepali). The participants were assured that their participation was voluntary, that their right to withdraw at any time would be upheld and that their collected information would remain confidential. All collected information was kept safely in a locked cabinet that could only be accessed by the researchers. The participants were also informed that all collected data would be destroyed after project completion. All study procedures involving human participants were conducted in accordance with the Declaration of Helsinki. The ethical approval code is SBRE-19-633. All of the participants provided informed consent to participate in the study before completing the online questionnaire.

## 3. Results

### 3.1. Sample Characteristics

A total of 361 South Asians provided informed consent and participated in the study. Nine respondents had missing data on more than 30% of the questionnaire items. Hence, 352 eligible participants were included in our analysis, of which 228 participants completed the questionnaire through online surveys, and 124 participants completed the questionnaire through face-to-face interviews.

The mean age of the participants was 38.92 years (SD = 13.42), and 59.7% were female. The participants’ countries of origin were India (35.8%), Pakistan (34.9%) and Nepal (29.1%). One fifth of the participants (20.5%) had an education level of primary school or lower, while 46.6% had received a secondary school education and 33.0% had received tertiary education or higher. More than half (51.4%) had a full-time or part-time job, and 57.9% had a monthly household income of HKD 20,000 or less (<USD 2564). The majority of participants adhered to a religion, including Islam (38.4%), Hinduism (29.0%), Sikhism (16.8%), Buddhism (7.7%) and Christianity (2.0%). Details of the participants’ characteristics are presented in Table 1.

### 3.2. Knowledge

The mean COVID-19 knowledge item score was 5.38 (SD = 1.45), indicating an overall correct response rate of 53.8% on the knowledge questionnaire. The correct response rates for the individual knowledge items ranged between 7.4% and 92.9%. Most participants provided correct answers to items regarding the signs and symptoms of COVID-19 (92.9%), maintaining social distance as an effective preventive measure (91.5%), the lack of a proven treatment or vaccine for the disease (86.6%) and the incubation period of SARS-CoV-2 (82.4%). However, the participants were less knowledgeable about topics such as whether wearing rubber gloves could effectively prevent infection (24.7%), whether only older people or those with underlying diseases are at a high risk of developing serious COVID-19 (20.2%), whether N-95 respirator masks should be routinely worn (14.2%) and whether the main mode of COVID-19 transmission is through contact (7.4%). The frequencies and associated percentages of participants who responded correctly to each item are presented in Table 2.

### 3.3. Attitudes

The mean perceived COVID-19 risk score was 3.41 (SD = 0.65) out of a maximum of 5, indicating that the participants generally perceived a mild level of disease risk (Table 3). Most participants agreed that COVID-19 is a serious disease (83.0%) and were fearful that they would become infected (61.9%) or be quarantined (69.0%). However, less than 40% of the participants thought that they or their family members were at risk of SARS-CoV-2 infection.

The mean perceived self-efficacy score with regard to controlling COVID-19 was 3.98 (SD = 0.70), out of a maximum of 5, which indicated an overall positive attitude regarding their competency in preventing self-infection. The majority of participants were confident that they could protect themselves against the disease (82.4%), and that COVID-19 would eventually be controlled in Hong Kong (87.8%).

### 3.4. Practices

The mean score for the practice of preventive measures was 3.50 (SD = 0.52) out of a maximum of 4, indicating that the participants often implemented the recommended preventive measures against COVID-19 (Table 4). The preventive measures most frequently implemented by the participants included wearing a surgical mask when using public transport or staying in a crowded venue (81.8%), avoiding non-essential travel outside Hong Kong (76.1%) and washing hands with liquid soap and water and rubbing with hand sanitiser for at least 20 s (71.9%).

### 3.5. Factors Associated with Knowledge, Attitudes and Practices

The results of our analysis of the factors that could affect participants’ KAP towards COVID-19 are presented in Table 5, Table 6 and Table 7. The knowledge of COVID-19 differed according to the participants’ education (F (2, 349) = 16.27, *p* < 0.001) and income levels (r = 0.23, *p* < 0.01). Participants with higher education and income levels were more likely to possess better knowledge about COVID-19. The participants’ perceptions of risk differed according to their marital status (F (1, 350) = 7.05, *p* = 0.008), education level (F (2, 349) = 6.69, *p* = 0.001) and work status (F (1, 350) = 10.84, *p* = 0.001). Participants who were married, had a higher education level and were unemployed tended to perceive a higher risk of COVID-19. The participants’ perceptions of self-efficacy were affected by their immigration status (F (1, 350) = 6.16, *p* = 0.014) and work status (F (1, 350) = 4.18, *p* = 0.042). Participants who were immigrants and being unemployed were more likely to have a higher level of confidence in their ability to avoid becoming infected. The practice of preventive measures differed by sex (F (1, 350) = 12.17, *p* = 0.001), education level (F (2, 349) = 9.99, *p* < 0.001) and income (r = 0.176, *p* = 0.001). Participants who were female and had higher educational and income levels were more likely to practice preventive measures.

The participants’ knowledge (r = 0.213, *p* < 0.01), perceived risk (r = 0.235, *p* < 0.001) and perceived self-efficacy (r = 0.388, *p* < 0.001) were positively correlated with the practice of preventive measures. Moreover, the associations of sex, educational level and attitudes with practices remained significant in the multivariate linear regression analysis. Participants who were male had a secondary school education or lower and who perceived a lower risk of becoming infected and lower self-efficacy in preventing themselves from becoming infected were less likely to implement preventive measures against COVID-19.

## 4. Discussion

To the best of our knowledge, this was the first study to investigate the KAP towards COVID-19 held by South Asian minority populations in Hong Kong. The results indicate that South Asian residents held inadequate levels of knowledge about COVID-19 prevention, perceived a mild level of risk related to COVID-19 infection and held positive attitudes towards controlling the spread of COVID-19. Most participants also complied with the recommended practices to prevent COVID-19 infections. By addressing these aspects within an ethnic minority population, our study expands on the knowledge generated by previous studies on KAP related to COVID-19, which were mainly conducted in general populations. This expanded understanding could inform strategies that could effectively reduce the susceptibility of ethnic minorities to COVID-19.

Our study yielded an overall correct response rate of 53.8% on the knowledge test among the participants. Most of the participants exhibited a basic understanding of the common signs and symptoms of COVID-19, the incubation period of SARS-CoV-2, the current disease treatment and the need for social distancing to prevent disease transmission. However, our participants expressed a lower level of knowledge about the transmission and risk factors of COVID-19. Approximately 50% of the participants did not know that an infected individual could spread the virus even when asymptomatic, and more than 90% were unaware of the fact that COVID-19 transmission occurred mainly through respiratory droplets and did not require direct contact with an infected person. Moreover, more than 80% of the participants did not realise that the risk factors leading to the substantial deterioration of health among COVID-19 patients included, but were not limited to, an older age and underlying comorbidities [14]. Although most participants recognised that social distancing could effectively prevent COVID-19, they held misconceptions about the essentiality of wearing rubber gloves and N-95 respirator masks in public places for disease prevention. In fact, the guidelines proposed by the Government indicate that members of the public do not need to wear rubber gloves and N-95 respirator masks during daily activities [15,16,17]. Such misconceptions may have led to the panic buying and unnecessary stockpiling of these products [18], which may jeopardise the supply of personal protective equipment for frontline medical workers. Therefore, further public education is warranted to rectify these misconceptions.

The participants’ overall correct response rate on the knowledge test was relatively low, compared with the overall correct response rates reported in studies conducted among the general populations in China (90% [6]) and Malaysia (80% [19]). However, the overall correct response rate in our study was comparable to that reported in an Iranian study (56% [20]). Differences in the education levels of the study populations might explain this discrepancy between studies. In our study and the Iranian study conducted by Honarvar et al. [20], the study participants generally had a lower education level, as demonstrated by the lower proportions of participants with a tertiary education relative to the population in the Chinese study [6]. This difference in education level may have resulted in disparities in the levels of health literacy possessed by the aforementioned populations and, consequently, their levels of awareness regarding COVID-19 and its prevention. Notably, although these studies assessed similar knowledge domains (i.e., symptoms, incubation, transmission and preventive measures), caution should be exercised when comparing the knowledge outcomes between these studies because of differences in the knowledge survey questions and scoring methods.

Our survey revealed that most participants perceived the serious nature of COVID-19 and were worried about its negative health effects. Still, less than 40% of the participants considered themselves or their family members at a high risk of COVID-19. The perception of disease risk was related to the marital status, education level and work status. COVID-19 can be rapidly transmitted within a family, and this may contribute to the higher level of risk perception observed among married individuals [21,22]. Similarly, individuals with a higher education level may have a better understanding of the disease, including its high infectivity and low death rate. They may automatically implement the necessary preventive measures to protect themselves, which may have alleviated their concerns about self-infection.

In our study, the participants displayed a positive attitude towards the prevention of COVID-19. More than 80% believed that they could protect themselves against the disease and that the pandemic would eventually be eradicated. Specifically, our results indicate that individuals who were immigrants tended to have a higher level of self-efficacy than those who were non-immigrants (born in Hong Kong). Compared with their home countries, i.e., India, Pakistan and Nepal, Hong Kong has a better healthcare system with greater resources, and the local government has implemented effective measures to control the spread of the disease (e.g., border closing, travel restrictions and compulsory isolation of suspected cases) [23]. These factors might have enhanced the immigrants’ self-efficacy in the ability of the local government to control the spread of COVID-19.

This study also assessed the levels of implementation of 10 COVID-19 preventive measures among the participants. A large proportion (87–94%) of the participants expressed that they usually complied with the key preventive measures advocated frequently by international and local authorities, including wearing a surgical mask, social distancing, performing hand hygiene, avoiding non-essential travel, avoiding touching animals or birds and following updates about the spread of the virus [1,2]. The high compliance with these measures implies that the key prevention messages have been successfully promulgated to the community. However, the participants appeared to have implemented certain measures less frequently, particularly practices related to the maintenance of toilet hygiene such as closing the toilet lid before flushing or pouring a sufficient amount of water into each drain outlet at home every week. Compared with other preventive measures, toilet hygiene appeared to have been less frequently promoted. Accordingly, more efforts should be made to educate the public about the importance of maintaining toilet hygiene to prevent the spread of the virus in households and communities. Generally, the participants’ frequent practice of the recommended preventive measures could partly be explained by the efforts made by the government to impose various prevention and control measures, such as the prohibition of group gatherings, during the COVID-19 pandemic.

We also determined that male participants with a lower education level and those with lower perceptions of risk and self-efficacy in preventing infection were less likely to implement the recommended preventive measures against COVID-19. Consistent with previous studies [6,19,24], men were less likely to take precautions against COVID-19. Individuals with a lower education level may have limited knowledge about the disease, which would limit their understanding of practices for the prevention of COVID-19. Finally, we demonstrated that the perceptions of risk and self-efficacy in preventing infection were associated with the level of implementation of preventive practices. This observation was consistent with the results of previous studies in which individuals were more likely to perform health-related behaviours if they perceived themselves as vulnerable to the disease, perceived the negative effects of the disease and perceived their self-efficacy to adopt behaviours to reduce these negative outcomes [25].

This study adopted Web-based and community-based approaches to the recruitment of South Asian residents of Hong Kong. Multiple studies have suggested that social media platforms could effectively recruit large samples of study participants to complete surveys during the pandemic [6,19]. However, members of ethnic minority populations may be underrepresented in these online surveys [26] because limited Internet access may restrict their participation [27]. Moreover, some ethnic minority residents with lower education levels may find it difficult to complete a self-administered online survey without assistance, which may lead to their withdrawal from the study prior to survey completion [27]. To address these issues, we adopted a community-based approach to participant recruitment, in addition to the Web-based approach, by providing the link to our collaborating organisations. We also arranged research assistants to help the participants complete the survey. These measures may have enhanced the rate of successful study completion, thereby limiting the exclusion of participants from the analysis due to incomplete survey responses [28].

This study had several limitations. First, the design involved both snowball and convenience sampling but only in Hong Kong, and therefore our findings cannot be generalised to South Asian ethnic minority populations in other countries. Second, the study sample was over-represented by female Pakistani participants as compared to the ethnic composition of South Asians suggested by the latest census in Hong Kong [9], which warrants caution in generalising the findings to the general South Asian population in Hong Kong. Third, this cross-sectional study investigated the participants’ KAP at a certain time point. Individuals’ perceptions and practices may vary according to the pandemic situation, and therefore longitudinal studies that examine the changes in KAP at various stages of the pandemic are recommended. Fourth, the participants’ KAP towards COVID-19 were examined primarily using a self-report approach, and no validated instruments were used to assess KAP. The lack of an objective measurement of the extent of the KAP examined in this study may have limited the reliability and comparability of the study findings. Finally, despite the fact that participants were assured of the anonymity and confidentiality of their responses in face-to-face interviews, social desirability bias may exist.

## 5. Conclusions

In conclusion, South Asian residents in Hong Kong held a basic level of knowledge about COVID-19, perceived a mild risk of the disease, had positive attitudes about its prevention and often implemented the recommended preventive measures. However, these individuals possessed certain misconceptions regarding the prevention of COVID-19 infection by using personal protective equipment (e.g., N-95 respirator masks and rubber gloves). To address these misconceptions, the local government and related authorities should devise and implement effective educational strategies to clarify these aspects. Culturally and linguistically appropriate health education could be developed to increase knowledge about the disease in South Asian populations and disseminated regularly through various social media, especially those who are less educated, and to encourage the implementation of necessary preventive measures. An increased awareness of COVID-19 prevention strategies would increase the competency of this population in terms of health maintenance during the current public health crisis. Ultimately, such strategies could effectively mitigate current increases in the number of confirmed COVID-19 cases in Hong Kong.

## Figures and Tables

**Table 1:** Sample characteristics (*n* = 352).

	Mean (SD)/*n* (%)
**Gender**	
Female	210 (59.7)
Male	142 (40.3)
**Age ^†^**	38.92 (13.42)
**Country of origin**	
Pakistan	123 (34.9)
India	126 (35.8)
Nepal	103 (29.3)
**Immigrant status**	
Non-immigrant	50 (14.2)
Immigrant	302 (85.8)
**Marital status**	
Single	62 (17.6)
Married	276 (78.4)
Divorced	9 (2.6)
Widowed	5 (1.4)
**Education level**	
Primary school or lower	72 (20.5)
Secondary school	164 (46.6)
University	84 (23.9)
Master or above	32 (9.1)
**Work status**	
Full-time	145 (41.2)
Part-time	36 (10.2)
Unemployed	82 (23.3)
Housewife	72 (20.5)
Student	9 (2.6)
Other	8 (2.3)
**Monthly household income (HKD)**	
<10,000	86 (24.4)
10,000–20,000	118 (33.5)
20,001–30,000	105 (29.8)
30,001–40,000	26 (7.4)
>40,000	17 (4.8)
**Religion**	
No	16 (4.5)
Hinduism	102 (29.0)
Islam	135 (38.4)
Sikhism	59 (16.8)
Buddhism	27 (7.7)
Christianity	7 (2.0)
Others	6 (1.7)

Data marked with ^†^ are presented as mean (standard deviation); all others are presented as frequency (%).

**Table 2:** Responses of knowledge items.

Item	Correctly Responded *n* (%)
1. The signs and symptoms of COVID-19 include fever, malaise, dry cough and shortness of breath.	327 (92.9)
2. There is currently no proved treatment or vaccine for this infectious disease, and the main treatment is supportive.	305 (86.6)
3. Only those who are older age or having underlying disease are at a high risk of deterioration into serious condition.	71 (20.2)
4. The main mode of COVID-19 transmission is through contact.	26 (7.4)
5. The time from exposure to onset of symptoms is typically around five days, but may range from two to fourteen days.	290 (82.4)
6. Members of the public should go out less and reduce social activities to maintain social distance with others as far as possible.	322 (91.5)
7. Wearing rubber gloves while out in public can effectively prevent infection.	87 (24.7)
8. Persons with COVID-19 cannot spread the virus to others when a fever is not present.	190 (54.0)
9. The symptoms of COVID-19 in children and adults are similar, but the symptoms in children are generally mild.	227 (64.5)
10. N-95 respirator should be routinely worn according to the current situation.	50 (14.2)

**Table 3:** Responses of attitude items.

Item	Disagree ^1^ *n* (%)	Uncertain *n* (%)	Agree ^2^ *n* (%)	Mean ^3^ (SD)
**Perceived risk of COVID-19**				3.41 (0.65)
1. I think COVID-19 is a serious disease.	37 (10.5)	23 (6.5)	292 (83.0)	3.99 (1.08)
2. I am fear of getting infected with COVID-19.	62 (17.6)	72 (20.5)	218 (61.9)	3.49 (1.01)
3. I am fear of getting quarantined if get infected.	77 (21.9)	32 (9.1)	243 (69.0)	3.51 (1.08)
4. My health will be severely affected if I get infected with COVID-19.	49 (13.9)	44 (12.5)	259 (73.6)	3.69 (0.95)
5. I think I will get infected with COVID-19.	88 (25.0)	130 (36.6)	134 (38.1)	3.07 (1.02)
6. I think my family will get infected with COVID-19.	94 (26.7)	129 (36.6)	129 (36.6)	3.02 (1.06)
7. I will not go to hospital even if I get sick because of risk of getting infected with COVID-19.	214 (60.8)	31 (8.8)	107 (30.4)	2.53 (1.18)
**Perceived self-efficacy in controlling COVID-19**				3.98 (0.70)
8. I believe I can protect myself against COVID-19.	25 (7.2)	37 (10.0)	290 (82.4)	3.88 (0.85)
9. I believe COVID-19 can finally be successfully controlled.	21 (6.0)	44 (12.5)	287 (81.5)	3.95 (0.85)
10. I have confidence that Hong Kong can win the battle against COVID-19.	20 (5.7)	23 (6.5)	309 (87.8)	4.11 (0.86)

^1^ including strongly disagree and disagree; ^2^ including strongly agree or agree; ^3^ scale ranged from 1 to 5. 1: strongly disagree; 2: agree; 3: uncertain; 4: disagree; 5: strongly agree.

**Table 4:** Responses of practice of preventive measures against COVID-19.

Item	Never *n* (%)	Sometimes *n* (%)	Often *n* (%)	Always *n* (%)	Mean ^1^ (SD)
1. Go out less and reduce social activities and maintain an appropriate social distance with others.	13 (3.7)	29 (8.2)	81 (23.0)	229 (65.1)	3.49 (0.80)
2. Avoid non-essential travel outside Hong Kong.	21 (6.0)	19 (5.4)	44 (12.5)	268 (76.1)	3.59 (0.84)
3. Avoid touching animals, poultry/birds or their droppings.	24 (6.8)	22 (6.3)	54 (15.3)	252 (71.6)	3.52 (0.89)
4. Wear a surgical mask when taking public transport or staying in a crowded place.	9 (2.6)	11 (3.1)	44 (12.5)	288 (81.8)	3.74 (0.64)
5. Perform hand hygiene before wearing and after removing a mask.	9 (2.6)	23 (6.5)	83 (23.6)	237 (67.3)	3.56 (0.73)
6. Wash hands with liquid soap and water and rub for at least 20 s.	3 (0.9)	20 (5.7)	76 (21.6)	253 (71.9)	3.64 (0.63)
7. Perform hand hygiene with 70 to 80% alcohol-based handrub if hand washing facilities are not available.	8 (2.3)	27 (7.7)	76 (21.2)	241 (68.5)	3.56 (0.73)
8. Put down the toilet lid before flushing	11 (3.1)	55 (15.6)	98 (27.8)	188 (53.4)	3.32 (0.85)
9. Pour half a litre of water into each drain outlet every week.	35 (9.9)	73 (20.7)	92 (26.1)	152 (43.2)	3.03 (1.02)
10. Follow the updates about the spread of the virus.	6 (1.7)	30 (8.5)	81 (23.0)	235 (66.8)	3.55 (0.72)
Overall practices					3.50 (0.52)

^1^ scale ranged from 1 to 4. 1: never; 2: sometimes; 3: often; 4: always.

**Table 5:** Univariate analyses of the associations of socio-demographic characteristics with knowledge, attitudes and practices.

	Knowledge		Perceived Risk		Perceived Self-Efficacy		Practices	
	M (SD)	F test	M (SD)	F test	M (SD)	F test	M (SD)	F test
**Sex**		1.35		3.34		3.78		12.17 **
**Male**	5.27 (1.57)		3.33 (0.62)		3.89 (0.82)		3.39 (0.59)	
**Female**	5.46 (1.36)		3.46 (0.66)		4.04 (0.60)		3.58 (0.44)	
**Immigrant status**		0.38		0.54		6.16 *		1.85
**Non-Immigrant**	5.36 (1.51)		3.40 (0.64)		3.94 (0.72)		3.49 (0.52)	
**Immigrant**	5.50 (1.02)		3.47 (0.69)		4.21 (0.57)		3.60 (0.48)	
**Marital status**		0.19		7.05 **		1.91		2.88
**Not Married**	5.45 (1.54)		3.24 (0.75)		3.88 (0.83)		3.42 (0.57)	
**Married**	5.37 (1.42)		3.46 (0.61)		4.01 (0.66)		3.53 (0.50)	
**Education**		16.27 ***		6.69 **		0.61		9.99 ***
**Primary school**	5.01 (1.19)		3.65 (0.62)		4.02 (0.79)		3.38 (0.51)	
**Secondary school**	5.12 (1.38)		3.38 (0.63)		4.00 (0.69)		3.44 (0.56)	
**University or above**	5.98 (1.51)		3.31 (0.56)		3.92 (0.66)		3.67 (0.41)	
**Work status**		3.58		10.84 **		4.18 *		0.41
**Unemployed**	5.23 (1.31)		3.53 (0.60)		4.06 (0.62)		3.49 (0.50)	
**Employed**	5.52 (1.55)		3.30 (0.67)		3.91 (0.77)		3.52 (0.53)	
		r		r		r		r
**Age**		−0.036		0.078		0.074		0.069
**Income**		0.233 **		−0.066		−0.007		0.176 **

* *p* < 0.05, ** *p* < 0.01, *** *p* < 0.001.

**Table 6:** Intercorrelation coefficients between the scores of knowledge, perceived risk, perceived self-efficacy, and practice.

	Knowledge	Perceived Risk	Perceived Self-Efficacy	Practice
**Knowledge**	1			
**Perceived risk**	0.044	1		
**Perceived self-efficacy**	0.095	0.428 ***	1	
**Practice**	0.213 **	0.235 ***	0.388 ***	1

** *p* < 0.01, *** *p* < 0.001.

**Table 7:** Multiple regression analysis on factors associated with preventive behaviors.

	Unstandardized Beta (se)	Standardized Beta	t	*p*
Female ^1^	0.140 (0.053)	0.133	2.62	0.009
Age	0.002 (0.002)	0.059	1.08	0.28
Married ^2^	0.081 (0.067)	0.065	1.21	0.23
Employed ^3^	0.034 (0.056)	0.033	0.61	0.54
Immigrant ^4^	0.084 (0.075)	0.057	1.12	0.26
Education level ^5^				
Primary school or lower	−0.282 (0.080)	−0.221	−3.54	<0.001
Secondary school	−0.181 (0.060)	−0.176	−3.04	0.003
Income	0.045 (0.025)	0.093	1.77	0.078
Knowledge	0.031 (0.018)	0.088	1.78	0.077
Perceived risk	0.087 (0.042)	0.110	2.08	0.038
Perceived self-efficacy	0.234 (0.038)	0.319	6.09	<0.001

^1^ male = 0, female = 1; ^2^ married = 1, single/divorced/widowed = 0; ^3^ full-time or part-time employment = 1, others = 0; ^4^ immigrant = 1, non-immigrant = 0; ^5^ the reference group was university or above.
